# Prognostic Biomarkers in Acute Coronary Syndromes: Risk Stratification Beyond Cardiac Troponins

**DOI:** 10.1007/s11886-017-0840-3

**Published:** 2017-03-17

**Authors:** K. M. Eggers, B. Lindahl

**Affiliations:** Department of Medical Sciences and Uppsala Clinical Research Center, Uppsala University, S-751 85 Uppsala, Sweden

**Keywords:** Acute coronary syndrome, Biomarkers, Risk prediction

## Abstract

**Purpose of Review:**

Cardiac troponin (cTn) plays an essential role for assessment of outcome in acute coronary syndrome (ACS). However, the prognostic value of cTn is not absolute. In this mini-review, we summarize the evidence on the utility of established biomarkers of left-ventricular dysfunction, hemodynamic stress, inflammation, and renal dysfunction for risk prediction beyond cTn in ACS.

**Recent Findings:**

Only few biomarkers consistently demonstrate additive prognostic value to cTn levels. The B-type natriuretic peptides (NPs) and growth-differentiation factor-15 (GDF-15) are most promising in this regard. However, there are uncertainties regarding the role of these biomarkers for guidance of treatment decisions, and their prognostic increment to cTn levels measured with high-sensitivity assays is largely unknown.

**Summary:**

The NPs and GDF-15 provide the strongest prognostic increment to cTn levels in ACS. However, the role of these biomarkers for clinical decision-making in contemporary settings has still to be defined.

## Introduction

Cardiac troponin (cTn) is released from cardiomyocytes exposed to ischemia severe enough to cause irreversible cell damage [1]. Measurement of cTn levels plays an essential role in the management of patients with suspicion of an acute coronary syndrome (ACS), and elevated levels are a prerequisite for the diagnosis of myocardial infarction (MI) [2]. In patients with symptoms of ACS, elevated cTn levels indicate the presence of an unstable coronary lesion and an increased risk of recurrent ischemic events. Higher cTn levels correlate with a larger MI size [3, 4] and, accordingly, greater mortality risk. cTn levels are for this reason used for both short- and long-term risk assessment, and cTn elevation is a commonly used clue for the selection of patients to beneficial therapies, e.g., coronary revascularization or anticoagulant treatment [5, 6].

**Fig. 1:**
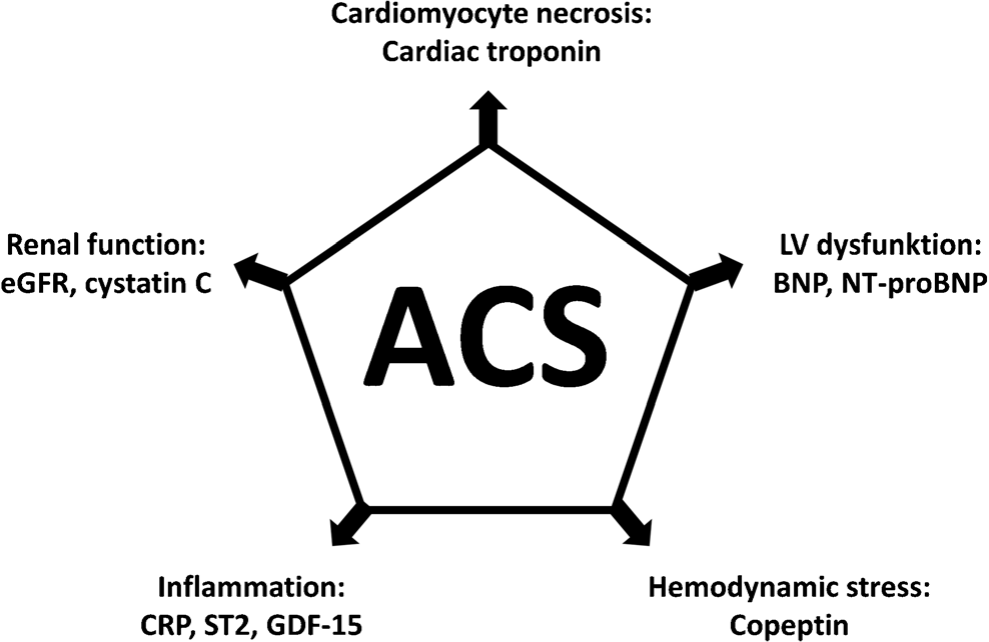
Pathobiologic mechanisms in acute coronary syndrome and associated biomarkers. *ACS* acute coronary syndrome, *LV* left-ventricular, *BNP* B-type natriuretic peptide, *NT-proBNP* N-terminal pro B-type natriuretic peptide, *CRP* C-reactive protein, *GDF-15* growth differentiation factor-15, *eGFR* estimated glomerular filtration rate

However, the prognostic value of cTn is not absolute. For example, high-risk patients with unstable angina usually do not have cTn elevation, and in ST-segment MI (STEMI), cTn levels may be affected by reperfusion modalities. Moreover, cTn levels do not cover all pathobiologic processes being relevant in ACS, e.g., left ventricular (LV) dysfunction, hemodynamic stress, inflammation, or renal dysfunction (Fig. 1). This has generated interest in prognostic biomarkers that can be used in ACS together with cTn for improvement of risk prediction. The past decades have seen a constant proliferation of such candidate biomarkers. In this mini-review, we aimed to summarize the evidence on this important topic. In order to provide information that is useful to the practicing clinician, we limit this review to established biomarkers that are measurable using currently marketed assays.

## Biomarkers of LV-Dysfunction—the Natriuretic Peptides

B-type natriuretic peptide (BNP) and its prohormone, N-terminal proBNP (NT-proBNP), are released from atrial and ventricular cardiomyocytes in response to myocardial stretch caused by volume or pressure overload. The physiologic actions of the natriuretic peptides (NPs) include natriuresis, vasodilatation, and inhibition of the effects of the renin-angiotensin-aldosterone system (RAAS) and the sympathetic nervous system. This counterbalances fluid retention and possibly prevents maladaptive forms of LV hypertrophy and fibrosis [7].

NP levels rise rapidly after myocardial ischemia as this often causes a transient increase in LV wall tension and myocardial stretch. The increases in NP levels in ACS are proportional to the size of the ischemic insult but also reflective of LV dysfunction during and prior to the current episode of ACS, important predictors of adverse outcome [8–13]. NP levels gradually decline during the course of weeks after ACS [14•]. In some patients, particularly those who eventually develop heart failure (HF), a second peak may occur after some days, reflecting adverse LV remodeling [8].

The NPs are powerful predictors of mortality and HF in ACS patients presenting with and without ST-segment elevation, independent of echocardiographic findings and cTn results [9–12, 15••, 16]. Whether this holds true even when cTn is measured using high-sensitivity (hs)-assays is at present uncertain. The NPs enhance the prognostic information obtained from the GRACE score [17, 18••] and are, moreover, useful predictors of functional LV recovery after ACS [13]. The association of the NPs with recurrent ischemic events in contrast is usually weak or non-existent in adjusted analyses. This seems surprising given the close relation of NP levels with the severity of coronary artery disease [19]. However, there is no causal link between NP levels and atherothrombosis which might explain this specific risk pattern.

The optimal timing of NP measurement for risk assessment is uncertain. Samples obtained late during the hospitalization or during follow-up are preferable to those obtained very early as they provide stronger associations with subsequent events [8, 14•, 16, 20]. However, whether or not temporal changes in NP levels might be associated with a change in prognosis is not clear [14•, 16, 20].

Several studies have investigated whether NP levels might identify patients with non-ST segment elevation ACS (NSTE-ACS) having particular benefit from an early invasive management. Although results from individual studies have been conflicting [9, 10], a recent meta-analysis including 8125 patients from five studies demonstrated a significant mortality reduction by an early invasive strategy in patients with “high” NP levels [21••]. The risk ratio was 0.74 (95% confidence interval [CI] 0.59–0.86), mainly driven by results from the FRISC-II study [10]. Regarding P2Y12 inhibitors, an analysis from the PLATO trial evaluating patients with all types of ACS failed to prove an interaction of NT-proBNP levels with the effect of ticagrelor treatment. However, NT-proBNP levels contributed to the information on the magnitude of the prognostic benefit of this drug [22].

As NP levels reflect the degree of LV dysfunction, it is conceivable to believe that they might be useful for guidance of pharmacological treatment improving LV remodeling. This has been investigated in a British study including 1725 ACS patients. In this analysis, a prognostic benefit from RAAS inhibitors emerged, however, only in patients with NT-proBNP levels in the highest quartile [23]. In the AVANT GARDE-TIMI 43 trial investigating 1101 ACS patients with elevated NP levels, no benefit of an early initiation of RAAS inhibition with valsartan, aliskiren, or their combination was seen compared with placebo [24]. In a prospective analysis of the MERLIN-TIMI 36 trial investigating 4543 NSTE-ACS patients, ranolazine, a drug with anti-ischemic effects, reduced the risk of adverse events in patients with BNP >80 ng/L but not in those with lower levels. In contrast to the hypothesis that ranolazine may have a specific effect on LV wall stress, it did not reduce BNP levels over time in that study [25].

## Biomarkers of Hemodynamic Stress—Copeptin

Copeptin is the c-terminal part of the prohormone of arginine-vasopressin and is released from the neurohypophysis in situations associated with endogenous stress [26]. Copeptin levels rise rapidly in multiple acute disorders including MI. However, copeptin is not cardiospecific. Besides MI, elevated levels can also be found in other cardiac diseases, e.g., myocarditis or HF but not in unstable angina [27]. Elevations are common in many non-cardiac diseases as well, e.g., stroke, sepsis, respiratory tract infections, or gastroesofageal reflux [26, 28].

Given its rapid increase in acute illnesses, copeptin has gained much interest as a tool for early ruling-out of MI [26, 27]. However, copeptin levels are also associated with LV dysfunction and remodeling, in a similar fashion as the NPs [29]. Accordingly, copeptin has been shown to be a strong predictor of mortality and HF in patients with ACS [29–31, 32••]. One of the first analyses evaluating the prognostic value of copeptin comes from the LAMP study enrolling 980 ACS patients, the majority having STEMI [30]. Copeptin levels independently predicted the combined endpoint of death or hospitalization for HF at 1 year (OR 2.33 [95% CI 1.55–3.49]), similarly as and additive to NT-proBNP. This indicates that the stimuli of the secretion of these biomarkers are different although both of them indicate hemodynamic stress. Similar as NT-proBNP, copeptin did not predict recurrent ischemic events in this analysis. Among 224 patients with acute MI and clinical HF from the OPTIMAAL study, higher copeptin levels outperformed BNP and NT-proBNP in predicting major cardiovascular (CV) events during 33 months of follow-up with a hazard ratio (HR) of 1.35 (95% confidence interval [CI] 1.05–1.72) [31].

While these studies did not consider cTn levels, more robust data come from the MERLIN-TIMI 36 trial [32••]. In this study, copeptin predicted the primary endpoint of CV death or HF at 1 year, independent of clinical factors, NT-proBNP, and cTnI measured with a sensitive assay (odds ratio [OR] 1.71 [95% CI 1.36–2.17]). Emerging data from studies in chest pain patients, however, indicate that the added prognostic value of copeptin to cTn levels may be upheld when these are measured with a hs-assay [33].

## Biomarkers of Inflammation

### CRP

C-reactive protein (CRP) is a phylogenetically highly conserved plasma protein and is part of the systemic response to inflammation [34]. CRP is an established marker for the detection of (bacterial) infections and various inflammatory and necrotic processes. CRP has been extensively studied as a marker for risk assessment in ACS in the last 20 years [35]. In patients with MI, CRP levels increase within 6 h after symptom onset and peak 2–4 days later [36]. CRP increases in response to myocardial injury and the maximum level is correlated to the size of the MI [37]. CRP has been detected in atherosclerotic plaques, but a causal relationship between CRP and coronary events is highly unlikely [38].

In most studies looking at the prognostic value of CRP in patients with NSTE-ACS, blood samples have been obtained on admission or at inclusion into randomized clinical trials. The first small study indicating that elevated CRP levels were associated with short-term CV events in NSTE-ACS patients was published 1994 [39], and thereafter, many studies have confirmed this association extending it to long-term follow-up [35]. The prognostic value of CRP regarding mortality is strong; the data regarding future MI are less convincing [40•]. In a meta-analysis based on 13 studies with 9787 patients and a total of 1364 adverse events (mainly death or a combination of death and MI), the pooled relative risks of long-term adverse outcomes were 1.40 (95% CI 1.18–1.67) for patients with CRP 3.1–10.0 mg/l and 2.18 (95% CI 1.77–2.68) for CRP >10.0 mg/l as compared to CRP ≤3.0 mg/l [35].

However, whether measurement of CRP contributes clinically relevant incremental information over and above clinical risk scores and other biomarkers is still controversial, and studies have shown contradictory results [15••, 41–44]. Notably, so far, no large study has been done including hs-cTn assays. Furthermore, there is no proven role for CRP for choice of treatment in ACS [45].

### ST2

ST2, a soluble interleukin‐1 receptor family member, is upregulated and secreted in response to mechanical stress [46]. It has been suggested that ST2 also is a marker of inflammation, fibrosis, and adverse myocardial remodeling [47, 48]. ST2 is only weakly correlated with other biomarkers, such as cTn and the NPs, indicating different modes of stimulation and release [49]. Hence, ST2 has been extensively studied in patients with HF and has been found to be strongly and independently associated with mortality [47]. The FDA has approved an assay for measurement of ST2 for prognostication in HF.

ST2 has been less extensively studied in ACS patients. ST2 has clearly no role for the diagnosis of MI [50]. However, it has consistently shown independent prognostic value both in studies in STEMI and NSTE-ACS regarding mortality or mortality/HF with HRs between 1.3 and 2.6 [49]. Unlike most other biomarkers, the prognostic value of ST2 is more frequently studied in STEMI than NSTE-ACS, and the HRs seem generally a little higher in studies of STEMI than NSTE-ACS. In some [51], but not all studies [32••, 52, 53], ST2 provided independent prognostic value also when other biomarkers such as cTn or NT-proBNP were entered in the multivariable models. In a recent study of over 1200 patients with STEMI, a biomarker combination consisting of ST2, cTnT, and myeloperoxidase provided incremental prognostic information to the TIMI STEMI risk score for the prediction of short-term risk of CV death or HF [54•]. Whether ST2 is useful for the selection of treatment in ACS is unknown. However, there is one study indicating that an elevated ST2 level identifies patients with MI that benefit from treatment with a mineralocorticoid receptor antagonist [55].

### Growth Differentiation Factor-15—GDF-15

GDF-15 is a member of the transforming growth factor β superfamily and is expressed in virtually all tissues, suggesting important general cellular functions, although its exact biological functions are still poorly understood [56]. GDF-15 is involved in regulating inflammatory and apoptotic pathways and is upregulated in many different pathological conditions including, but not restricted to, CV diseases [56].

GDF-15 levels have consistently been shown to be strong and independent predictors of mortality and disease progression in patients with ACS. A meta-analysis including 8903 patients from eight ACS studies found a pooled HR of 1.66 (95% CI 1.47–1.87) regarding the combined endpoint of death or MI [57]. In this analysis, GDF-15 >1200 ng/L indicated a low risk and GDF-15 >1800 ng/L indicated a high risk for future CV events.

In the large PLATO trial evaluating 16,876 ACS patients followed for 1 year, higher levels of GDF-15 at study inclusion were associated with raised risks of mortality (HR 1.41 [95% CI 1.31–1.53]), spontaneous MI (HR 1.15 [95% CI 1.05–1.26]), and stroke (HR 1.19 [95% CI 1.01–1.42]) as well as of all types of major non-CABG-related bleeding (HR 1.37 [95% CI 1.25–1.51]) [58••]. These risk estimates were independent of clinical factors and levels of hs-cTnT, cystatin C, CRP, and NT-proBNP. Similar findings emerged from a subanalysis of the PROVE IT-TIMI 22 study investigating 3501 ACS patients followed for 2 years. In that analysis, GDF-15 levels measured at hospital discharge were significantly associated with the risk for death or MI independent of clinical factors, BNP, and CRP [59]. In another study in NSTE-ACS patients, GDF-15 together with NT-proBNP provided the strongest increment to the GRACE risk score [18••].

In the FRISC-II trial randomizing patients with NSTE-ACS to an invasive or conservative strategy with a follow-up for 2 years, elevated GDF-15 levels independently predicted the risk of death or MI in the conservative group but not in the invasive group. The interaction between GDF-15 levels and the treatment strategy was statistically significant in this study [60]. These results indicate that GDF-15 might be useful for the selection of patients for invasive treatment. Somewhat in contrast to the lack of prognostic value of GDF-15 in invasively treated patients in FRISC-II, the addition of levels of GDF-15, NT-proBNP and the extent of coronary artery disease to clinical variables in a study of 5174 revascularized patients with NSTE-ACS from the PLATO trial independently improved prognostication of CV death or spontaneous MI (c-statistics 0.69 for the full model vs 0.65 for the clinical model) [61].

## Biomarkers of Renal Function

### Estimated Glomerular Filtration Rate

ACS patients with chronic kidney disease (CKD) represent a particularly vulnerable group. The correct estimation of renal function is crucial for risk prediction in these patients, for decision on treatment strategies and guidance of pharmacological dosing. However, the glomerular filtration cannot be measured directly. In daily practice, the serum creatinine concentration or creatinine-based equations are used as surrogates. Serum creatinine is unfortunately an unreliable estimate as it is insensitive for the detection of moderate reductions in renal function and affected by factors such as age, gender, muscle mass, physical activity, and diet.

The most commonly used equations to calculate the estimated glomerular filtration rate (eGFR) are the Modification of Diet in Renal Disease (MDRD) study formula and the Cockcroft-Gault (CG) formula [62, 63]. There is a continued debate on which of them is most accurate. None was developed or validated in patients with cardiac disease. In addition, they differ in variables and coefficients. This results in lower eGFR calculated by the CG formula in the elderly and subjects with lower body mass index, cohorts known to be at higher risk during and after ACS [64, 65]. Accordingly, the CG formula has been shown to be superior for risk prediction compared to the MDRD formula as it classifies more patients subsequently having worse outcome to a lower renal function stage [64, 65]. Recently, the Chronic Kidney Disease-Epidemiology equation has been introduced as a more exact estimate of the eGFR compared to the other formulas [66]. Results from the PLATO trial suggest that it also more closely mirrors the risk of adverse events in ACS [67••].

Regardless of the applied equation, ACS patients have a continuous increase in mortality risk along with decreasing eGFR [15••, 64, 65, 67••, 68]. The association with recurrent ischemic events is weaker and often abrogated following adjustment for clinical factors. Notably, most of these studies are based on clinical trials in which patients with severe CKD or dialysis had been excluded.

Only few studies have investigated the added prognostic value of the eGFR to cTn levels [15••, 67••, 68]. This likely reflects the fact that renal function assessment is an integral part of the management of patients with ACS. While these studies demonstrated an added prognostic value of the eGFR to cTn, the magnitude of this effect seems only to be moderate [67••].

The prognostic interrelation between cTn and the eGFR is complicated by the fact that small cTn elevations are common in subjects with severe CKD. This has been attributed to myocardial wall stretch secondary to volume overload, chronic myocardial ischemia, and cardiotoxicity due to changes in osmolarity or ion fluxes [69, 70]. Impaired renal clearance of immunoreactive cTn fragments also contributes to cTn elevation, in particular in patients with more advanced CKD stages [71]. These prognostically less adverse mechanisms together with a higher prevalence of other adverse comorbidities contribute to a nonlinear relationship in the relative risk associated with cTn levels along with decreasing eGFR: despite higher absolute event rates and higher cTn levels in more severe CKD, the relative risk estimates for cTn tend to be lower compared to mild or moderate CKD stages [72].

### Cystatin C

The concerns regarding the various eGFR equations have directed investigators to search for other, more reliable markers of renal function. One of them is cystatin C, a 13-kDa protein that is constantly produced in all nucleated cells and filtered by the renal glomeruli without secretion or subsequent reabsorption to the blood flow [73]. Cystatin C is considered to be more sensitive than serum creatinine and at least as sensitive as eGFR equations for the estimation of renal dysfunction [74]. Additional interest in cystatin C arises from the fact that it is an inhibitor of cytokine-activated elastolytic cysteine proteases that are involved in the promotion of atherosclerotic plaques and their destabilization [75]. Accordingly, cystatin C appears to reflect different pathways in ACS which hypothetically might augment its prognostic value compared to other estimates of renal function.

Cystatin C has been shown to be an independent predictor of mortality in ACS. In some but not all studies, cystatin C was a stronger predictor of adverse outcome than serum creatinine or the eGFR [76–78]. In the so far largest study, a subanalysis from the PLATO trial (16,401 ACS patients), the c-statistics of cystatin C regarding the combined endpoint of CV death or MI at 1 year was 0.69, similar as for the MDRD and CG equations (C-statistics 0.69 for both). However, there was no incremental prognostic value of cystatin C when added to a clinical model including cTnI, hs-CRP, and NT-proBNP [67••].

## Summary and Outlook

There is an intense search for CV biomarkers that might add to the prognostic information obtained from cTn levels, and many of these biomarkers have been suggested for clinical use. However, there are still gaps in knowledge. Surprisingly, few studies investigated such biomarkers in the context of cTn results obtained with hs-assays. This leaves the question whether positive results, in particular from older studies hold true in contemporary settings where hs-cTn assays are increasingly used. Although many of the biomarkers described in this mini-review may complement existing methods for outcome prediction in ACS patients, there are only few head-to-head comparisons. This kind of studies is important for clinicians to appreciate the prognostic value of any individual biomarker in a broader context. Results from the PLATO and MERLIN-TIMI 36 studies suggest that biomarkers of inflammation and renal function only provide modest predictive value when clinical factors and cTn results are taken into consideration. The NPs and GDF-15 are much stronger risk indicators [22, 32••, 44, 67••] and have also been shown to add considerable prognostic increment to the GRACE score [18••]. Table 1 presents an overview regarding the clinical utility of the biomarkers summarized in this mini-review.

**Table 1:** Prognostic biomarkers in acute coronary syndrome

Biomarker	Prognosis			Selection of therapy
	Death	MI	Heart failure	
cTn	+++	++	++	++
BNP/NT-proBNP	+++	-	+++	+
Copeptin	++	-	++	-
CRP	++	-	-	-
ST2	++	-	++	-
GDF-15	+++	+	+	+
eGFR	++	-	+	-
Cystatin C	++	-	+	-

*MI* myocardial infarction, *cTn* cardiac troponin, *BNP* B-type natriuretic peptide, *NT-proBNP* N-terminal pro B-type natriuretic peptide, *CRP* C-reactive protein, *GDF-15* growth differentiation factor-15, *eGFR* estimated glomerular filtration rate

However, even a strong association with outcome is seldom enough for a biomarker to be clinically useful. A biomarker should also provide clinical information of such magnitude that it directly affects the clinical management of each individual patient. Accordingly, studies consistently supporting the benefit of specific therapeutic interventions to modify the risk associated with higher biomarker levels are needed to better define the utility of such a biomarker. Such studies should also investigate whether a reduction in risk goes hand in hand with a reduction in biomarker levels.

Even though data on numerous candidate biomarkers have been published in the literature, they have a long way to go before their clinical routine use can be recommended. This leads to the question whether other approaches for improvement of outcome prediction, not necessarily focusing on single biomarkers, might be more useful. There has been a rapid development of technologies for proteomic studies observing 1000–5000 proteins [79]. In analogy with genome-wide association studies, hypothesis-free association studies are underway to identify single biomarkers or biomarker clusters being representative of distinct pathways in ACS [79, 80]. The assessment of microRNAs is another option that has gained increasing interest. MicroRNAs are small non-coding RNAs that are key regulators of complex biological processes involved in the development of various CV conditions. Accumulating evidence suggests a potential role for microRNAs as diagnostic tools in ACS and possibly, as prognostic indicators [81]. A different, more clinically oriented approach to maximize the wealth of prognostic information obtained from biomarker results relies on their integration into more complex predictive models [82, 83]. This overcomes the limitations inherent to any single biomarker and such approaches are increasingly used in CV medicine.

## Conclusion

Although the proliferation of new prognostic biomarkers in ACS has been remarkable in recent years, only few of them have convincingly proven incremental value to cTn results. While the NPs and GDF-15 are most promising in this regard, their role in clinical decision-making has yet to be defined, in particular, with respect to biomarker-guided treatment decisions in contemporary settings where hs-cTn assays are used.
